# The Sun Through Time

**DOI:** 10.1007/s11214-020-00773-9

**Published:** 2020-12-08

**Authors:** Manuel Güdel

**Affiliations:** Department of Astrophysics, University of Vienna, Türkenschanzstr. 17, 1180 Vienna, Austria

**Keywords:** Stellar activity evolution, Stellar wind evolution, Stellar mass-loss evolution, Stellar rotation, Stellar spin-down

## Abstract

Magnetic activity of stars like the Sun evolves in time because of spin-down owing to angular momentum removal by a magnetized stellar wind. These magnetic fields are generated by an internal dynamo driven by convection and differential rotation. Spin-down therefore converges at an age of about 700 Myr for solar-mass stars to values uniquely determined by the stellar mass and age. Before that time, however, rotation periods and their evolution depend on the initial rotation period of a star after it has lost its protostellar/protoplanetary disk. This non-unique rotational evolution implies similar non-unique evolutions for stellar winds and for the stellar high-energy output. I present a summary of evolutionary trends for stellar rotation, stellar wind mass loss and stellar high-energy output based on observations and models.

## Introduction

Solar activity was recognized as an important property of the Sun in observations of magnetic fields in sunspots (Hale 1908), the occurrence of giant outbursts initially in white light (Carrington 1859; Hodgson 1859), the presence of a very hot (millions of K) corona initially in optical lines (Grotrian 1939; Edlén 1942), and later in radio waves (Hey 1946) and X-rays (Burnight 1949). We now know that magnetic fields are at the origin of all these phenomena, as they can store energy derived from convective motions at and below the photospheric level, but can also continuously or episodically release energy in higher atmospheric layers (the chromosphere, the transition region, the corona, and the solar-wind region).

Magnetic fields originate in the dynamo deep inside the convection zone; differential rotation and convective motion act together to amplify magnetic fields that rise buoyantly to the surface where they form magnetic active regions with “magnetic loops” reaching out into the corona. A solar coronal wind was inferred initially from observations of comets (Biermann 1951) and explained theoretically by Parker (1958), followed by *in situ* observations by space probes in the early 1960s.

In what sense solar/stellar rotation itself matters for “magnetic activity” was less clear; it was stellar astronomy that made important contributions to this question. Kraft (1967) reported that the average rotational velocities of stars with strong Ca ii emission are higher than velocities for weak Ca emitters. This pointed to an evolutionary spin-down of stars because stronger Ca ii emitters are younger; Kraft (1967) suggested that magnetically coupled winds like the solar wind are responsible for the angular momentum loss and associated this process with outer convective layers in cool stars. The angular momentum loss process was theoretically explained by Weber and Davis (1967) via magnetic wind torques. In another seminal paper, Skumanich (1972) laid the foundations of subsequent stellar statistical studies of age, evolution of activity, and rotation. Skumanich suggested that Ca ii emission declines with the inverse square root of the age, and the same decay law should also hold for the rotation velocity and the surface magnetic field strength. These observations were the foundation of age-rotation-activity relations that have been quantitatively refined until the present time.

Apart from the role it plays for stellar structure, atmospheres and spin-down, stellar activity has been recognized as a key parameter for the evolution of planets. Short-wavelength radiation (ultraviolet [UV], extreme ultraviolet [EUV], and X-rays) affects the thermal structure of planetary atmospheres, can drive chemical reactions, and can lead to escape and erosion of planetary atmospheres. But also the stellar wind, shock fronts and high-energy particles it transports interact with planetary outer atmospheres and – if present – magnetospheres; a variety of interactions can again lead to chemical reactions, heating, and atmospheric loss.

Understanding the evolution of stellar magnetic fields and magnetic activity as expressed in high-energy radiation and winds is therefore pivotal if we are to understand the long-term evolution of planets. In fact, an observation of an exoplanet provides a snapshot in time but cannot by itself explain the composition and structure of an atmosphere. Comprehensive evolutionary models of the host star are required to trace the evolutionary processing of atmospheres, for rocky planets complemented with evolutionary models of the planetary interiors (convection, cooling, outgassing and ingassing).

The following subsections discuss the long-term evolution of stellar rotation and spin-down, winds, and high-energy (UV/EUV/X-ray = XUV) radiation with the aim of summarizing trends in the evolution of this stellar output. Stellar ages themselves come therefore into the discussion, but the intent here is not to provide means to estimate stellar ages (see Mamajek and Hillenbrand 2008 for a discussion of stellar ages and their relation with other stellar parameters). Many of the properties discussed below in fact evolve non-uniquely within an age range that is crucial for the setup of planetary atmospheres and their potential habitability, i.e., up to several 100 Myr after star/planet formation.

## Stellar Rotation

Stellar rotational evolution has been the subject of many publications aiming at studying basic physical concepts and testing them with observations (e.g., Gallet and Bouvier 2013). We briefly discuss here a model that includes all key ingredients for a semi-empirical description of stellar spin-down (Johnstone et al. 2015).

Observationally, young open clusters (i.e., co-eval samples of stars) show a large spread in rotation periods for any given stellar mass. Most likely, these stars were starting their rotational evolution with different initial conditions (which we take to be the rotational state after the dispersal of protostellar/protoplanetary disks a few million years after the formation of a protostar). In the pre-main sequence stages, stars still contract and therefore spin up until they reach the zero-age main sequence. After that time, stars can only spin down as a magnetized wind removes angular momentum. Interestingly, after about 700 Myr of evolution for solar-mass stars, they all end up at a rotation period nearly uniquely determined by the stellar mass and age (Soderblom et al. 1993). This *convergence* is ascribed to a feedback between angular momentum removal by a magnetized coronal wind and the rotationally induced operation of an internal dynamo that generates the magnetic fields. The close relation between magnetized and ionized winds and stellar coronae suggests that the wind mass-loss rate also declines with time.

To understand evolutionary spin-down, we therefore need knowledge on the initial stellar rotation rate, $\Omega _{0}$, the internal structure of the star (from stellar-structure models), and the rate at which angular momentum is removed from the star by the wind. To discuss the basic physical ingredients, I use the example of Johnstone et al. (2015) although other models have been presented in the literature (e.g., Gallet and Bouvier 2013, see also the physical wind model by Airapetian and Usmanov 2016). The model of Johnstone et al. (2015) consists of a system of equations with some free parameters that need to be fitted to the observed rotational distributions of stellar clusters. It was initially applied to stars with a radiative core and a convective envelope in the mass range of 0.4–1.1 $M_{\odot }$ including core-envelope coupling torques, but was recently extended to include also fully convective stars down to 0.1 $M_{\odot }$ using a different torque formula (Johnstone et al. 2020).

We denote with $\Omega _{*}(t)$ the angular rotation rate, $\dot{M}(t)$ the wind mass-loss rate and $B(t)$ the surface magnetic field strength. The magnetic torque acting on the star, $\tau $, is a function of stellar mass $M_{*}$, radius $R_{*}$, $\dot{M}$, $B$, and $\Omega _{*}$,

1 $$ \tau = f(B, \dot{M}, M_{*}, R_{*}, \Omega _{*}) \approx B^{0.87} \dot{M}^{0.56}R_{*}^{2.87}\Omega _{*} $$

where the numerical example on the right-hand side is simplified from a torque formula derived from MHD simulations by Matt et al. (2012). The spin-down rate then is,

2 $$ \frac{{\mathrm{d}}\Omega _{*} }{{\mathrm{d}}t} = \frac{1}{I_{*}}\left (\tau - \frac{{ \mathrm{d}}I_{*}}{{\mathrm{d}}t}\Omega _{*}\right ), $$

where $I_{*}$ is the star’s moment of inertia from stellar structure models. We next assume

3 $$ \dot{M} = \dot{M}_{\odot } \left (\frac{R_{*}}{R_{\odot }}\right )^{2} \left (\frac{\Omega _{*}}{\Omega _{\odot }}\right )^{a} \left (\frac{M_{*} }{M_{\odot }}\right )^{b} $$

where the exponents $a$ and $b$ are fitted to observational constraints. Vidotto et al. (2014) derived the equatorial magnetic field strength of the dominant dipole component from observations as

4 $$ B = B_{\odot }\left (\frac{\Omega _{*}\tau _{*}}{\Omega _{\odot }\tau _{ \odot }}\right )^{1.32} $$

where $\tau _{*}$ is the convective turnover time of the star. $B$ “saturates” for rapidly rotating stars, i.e., is no longer a function of $\Omega _{*}$, which means that $\dot{M}$ should also saturate. The rotation rate where saturation sets in can be described by

5 $$ \Omega _{*, \mathrm{sat}}(M_{*}) = \Omega _{\mathrm{sat}}(M_{\odot })\left ( \frac{M_{*} }{M_{\odot }}\right )^{c} $$

where $c$ is a further fit parameter, and $\Omega _{*, \mathrm{sat}}(M_{\odot }) \approx 15~\Omega _{\odot }$.

The above equations can be solved once the free parameters $a$, $b$, and $c$ have been fitted to rotation distributions for different ages, and initial conditions for $\Omega (t_{0})$ are assumed, using observed $\Omega _{*}$ distributions of clusters in star-forming regions.

**Fig. 1:**
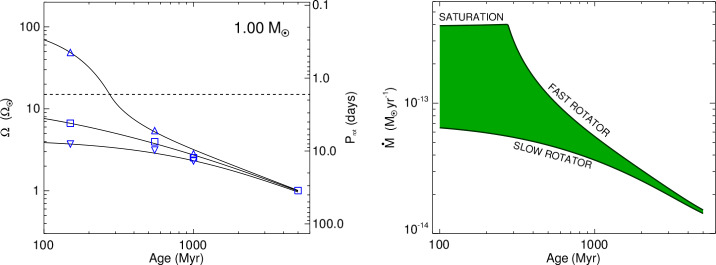
Left: Modeled rotational evolution tracks of a 1 $M_{ \odot }$ star at the 10th, 50th, and 90th percentiles of the rotational distribution, shown from 100 Myr to 5 Gyr. The blue symbols mark the same percentiles from observational distributions. The dashed line indicates the limiting rotation rate above which the wind mass-loss rate and the surface magnetic field saturate. – Right: The evolution of the solar wind mass-loss rate with age on the main sequence. The green area includes all possible $\dot{M}$ evolutionary tracks for different initial rotation periods at the 10th percentile (bottom edge) and the 90th percentile (top edge). The fastest rotators remain saturated at a maximum $\dot{M}$ level during the first 300 Myr. (From Johnstone et al. 2015.)

Fig. 1 shows examples for the spin-down evolution of a solar-mass star with different initial rotation rates, as well as the required wind mass-loss history from the same model. As required by the observations, the rotation rates converge to a unique age-dependent value after ∼700 Myr (where the width of the $\Omega $ distribution between the 10th and the 90th percentiles is ∼0.2 dex; Johnstone et al. 2015; Soderblom et al. 1993), and the same must apply for $\dot{M}$ as the primary cause for spin-down. After $\sim 700$ Myr, the mass-loss rate declines in time $t$ roughly as

6 $$ \dot{M} \propto t^{-0.75}. $$

It relates to the rotation period as

7 $$ \dot{M} \propto R_{*}^{2}~M_{*}^{-3.36}~\Omega _{*}^{1.33} $$

i.e., $\dot{M}$ decreases strongly with stellar mass but increases with rotation velocity; the saturation mass-loss rate is

8 $$ \dot{M}_{\mathrm{sat}} = 37 \dot{M}_{\odot } \left (\frac{M_{*}}{M_{\odot }} \right )^{1.3}, $$

showing that higher-mass stars can achieve higher mass-loss rates. Saturation of $\dot{M}$ limits the mass-loss rates of young, very rapid rotators, as shown in Fig. 1.

Because of the widely distributed initial rotation rates after star formation, the $\dot{M}$ and $\Omega _{*}$ histories are non-unique. We see that $\dot{M}$ of solar analogs is distributed over nearly an order of magnitude (10th to 90th percentile, green area) near the Zero-Age Main-Sequence (ZAMS) age (Fig. 1-right; Johnstone et al. 2015).

## Stellar Winds

Stellar winds have already been described above, together with stellar magnetic fields, as the key agent for stellar spin-down. What is the observational situation? Stellar winds are very difficult to detect directly owing to their weak emission; however, a few observational methods have been developed to infer the mass-loss rate $\dot{M}$ from other features that are controlled by the stellar wind. Observational wind detection/measurement methods can therefore be classified as *direct* and *indirect*.

*Direct wind measurements* make use of electromagnetic emission of the wind plasma itself, or optical-depth related absorption or attenuation in the wind. *Indirect wind measurements* use features for which the wind is responsible but that themselves are distinct from the wind.

We briefly summarize methods described in the literature and provide some key results for each one.

### Direct Wind-Detection Methods

#### Bremsstrahlung at Radio Wavelengths

Because winds from cool stars are ionized, they emit bremsstrahlung as a consequence of accelerating/decelerating interactions between electrons and ions. The bremsstrahlung emissivity is a function of electron temperature, $T$, and number density, $n_{\mathrm{e}}$. The radio free-free flux spectrum for an optically thick, constant-velocity, fully ionized isothermal spherical (isotropic) wind follows from

9 $$ S_{\nu } = 9 \times 10^{10} \ \left (\frac{\dot{M}}{v}\right )^{4/3} T^{0.1} \ \nu ^{0.6} \ d^{-2}\ \text{mJy}, $$

(Panagia and Felli 1975; Wright and Barlow 1975; Olnon 1975). Radio observations over a wide range of frequencies can therefore provide detections or upper limits for $\dot{M}$ given a wind temperature and a terminal wind velocity, $v$. Searches best involve high radio frequencies or millimeter wavelengths (Doyle and Mathioudakis 1991; Mullan et al. 1992), but interesting upper limits have been reported also for centimetric wavelengths, such as an upper limit for the evolved late F-type star Procyon translating to $\dot{M} < 2\times 10^{-11}~M_{\odot }\,{\text{yr}}^{-1}$ (Drake et al. 1993). Initially, the method was often used for radio-loud nearby M dwarfs, for which Lim and White (1996), Lim et al. (1996), and van den Oord and Doyle (1997) derived $\dot{M} <$ a few times $10^{-10}~M_{\odot }$ yr^−1^, down to $\lesssim 7\times 10^{-12}~M_{\odot }$ yr^−1^ for a wind temperature of $10^{4}$ K or $10^{6}$ K, respectively. Turning to solar-mass stars, Gaidos et al. (2000) and Fichtinger et al. (2017) studied an age sequence of solar analogs at 6–14 GHz but found only upper limits for the radio flux corresponding to wind mass-loss rates of $\dot{M} < 3\times 10^{-11}~M_{\odot }\,{\text{yr}}^{-1}$. The latter authors constrained the ZAMS mass of the Sun to no more than 1.02 times the present mass, which rejects a popular explanation of the Faint Young Sun Paradox (FYSP). The FYSP states that geological evidence implies mild climates on early Earth and Mars even though the solar luminosity was only $\approx 70-75\%$ of the present level. A slightly more massive Sun would have been more luminous in the early main-sequence stages, and therefore the Sun could have been brighter than in the standard evolution model. To solve the FYSP using an Earth-like or CO_2_ atmosphere, a mass excess of $>2$% is required at the ZAMS.

#### Radio Free-Free Optical Depth

A sufficiently dense wind can become optically thick to underlying stellar radio emission. If the optically thick surface is located above the lower coronal level, then radio flares would not be seen on active stars, in contrast to observational evidence. From the condition of small optical depth, Lim and White (1996) obtained $\dot{M} \lesssim 5\times 10^{-14} - 10^{-12}~M_{\odot }$ yr^−1^ for $v = 300-600~\text{km}\,\text{s}^{-1}$ and wind temperatures of $10^{4}-10^{6}$ K for the dMe star YZ CMi. For solar analogs we are focusing on here, Fichtinger et al. (2017) derived upper limits at different ages confirming their upper limits from bremsstrahlung emission (see above).

#### Radio Wave Propagation

Radio waves can propagate only if their frequencies exceed the local electron plasma frequency that itself depends on $n_{\mathrm{e}}^{1/2}$ ($n_{\mathrm{e}}$ being the electron number density). If a planet is supposedly a magnetospheric radio source, non-detections of this radiation can set upper limits to the mass-loss rate given a wind velocity. Vidotto and Donati (2017) estimated $\dot{M} \lesssim 10^{-10}~M_{\odot }$ yr^−1^ for the T Tauri star V830 Tau and a close-in hot Jupiter.

#### Radio Waves from Shocks in Winds

Coronal eruptions during large magnetic reconnection events can produce *coronal mass ejections (CMEs)* that form shocks which travel through the interplanetary space. Particles accelerated in these shock fronts induce radio radiation at the plasma frequency (or its harmonic) and could therefore be detected. This radiation is well known as Type-II radio emission in the solar corona. In very active stars, the CME mass loss rate maybe an important contributor to the total mass loss rate (Khodachenko et al. 2007), but observationally stellar Type-II bursts remain elusive (Crosley and Osten 2018).

#### X-Ray Emission

A hot ($\approx 10^{6}$ K) ionized wind produces continuum and line emission in the X-ray regime, like stellar coronae but presumably at much lower flux levels. Because thermal X-ray emission scales with $n_{\mathrm{e}}^{2}V$ where $V$ is an emitting volume, the observable emission should be stronger for higher wind mass-loss rates (given a fixed velocity), and the emission should come from close to the star. A detection is difficult because the emission from active coronal regions outshines wind emission. Lim and White (1996) estimate $\dot{M} \lesssim 6\times 10^{-11}~M_{\odot }$ yr^−1^ for the active mid-M dwarf YZ CMi, but generalizing such estimates to other active stars including detected solar-mass stars (taking the detected X-ray luminosity as an upper limit) results in upper limits in the range of $\dot{M} < 10^{-12} - 10^{-10}~M_{\odot }$ yr^−1^.

#### Astrospheric Charge Exchange

Neutral hydrogen from the interstellar medium can penetrate the stellar wind region (the “astrosphere”) and induce charge exchange (CX) with the heavy wind ions. Such reactions can lead to line emission in the X-rays. This method is very challenging, requiring a nearby star and a very low general X-ray background; it has been applied only once, to the M dwarf Proxima Centauri; Wargelin and Drake (2001) and Wargelin and Drake (2002) estimated the expected emission and specifically investigated high-resolution X-ray images around Prox. Cen for excess CX emission from the astrosphere. They found none, setting an upper limit of $\dot{M} < 3\times 10^{-13}~M_{\odot }\,{\text{yr}}^{1}$ for the total wind mass loss rate.

### Indirect Wind-Detection Methods

#### Ly$\upalpha $ Absorption in the Astrospheric Hydrogen Wall

The interaction between the wind-blown ionized “astrosphere” around a star (equivalent to the heliosphere, the latter with a radius of order 120 au) with a partially neutral interstellar medium leads to charge exchange interactions and a pile-up of hot neutral hydrogen in the heliopause region. This “hydrogen wall” produces excess absorption in the stellar Ly$\upalpha $ profile that can be related to a wind mass-loss rate based on complex numerical simulation models. More Ly$\upalpha $ absorption essentially means higher $\dot{M}$. This method was pioneered by B. Wood an colleagues (see review by Wood 2004) and has succeeded in obtaining $\dot{M}$ values for nearly 20 stars, many of them having masses similar to the Sun’s although some M dwarfs were also included. They infer a correlation with the average coronal X-ray surface flux of the form $\dot{M} \propto F_{\mathrm{X}}^{1.34\pm 0.18}$ and a wind decay law

10 $$ \dot{M}(t) \propto t^{-2.33\pm 0.55}, $$

for G and K-type stars. Interestingly, however, they find only low upper limits for $\dot{M}$ for active, young stars ($\lesssim 700$ Myr for solar analogs). It is unclear if this is related to specific magnetic configurations on the star that lead to wind suppression (e.g., large dipolar fields), or polar outflows instead of spherical winds; the breakdown of winds is problematic for the observed spin-down, however (Johnstone et al. 2015).

#### Wind-Planet Interactions

Stellar winds interact with planetary outer atmospheres and magnetospheres in diverse processes (ion escape, sputtering, charge exchange etc, see Lammer et al. 2003). They also transport shocks and high-energy particles that can ionize and chemically modify upper atmospheres (e.g., Airapetian et al. 2016). The collision between the wind and a planetary magnetosphere can form a detectable shock in front of the planet (Vidotto et al. 2011a). Several estimates have been made for the wind density or mass-loss rates based on observed spectral features. Kislyakova et al. (2014) estimated, from excess Ly$\upalpha $ absorption during a transit of the hot Jupiter, $\dot{M}= 4\times 10^{-14}~M_{\odot }$ yr^−1^ for the near-solar analog (F9 V-type) HD 209458. Lecavelier des Etangs et al. (2012) similarly found $\dot{M} \approx 5\times 10^{-15}~M_{\odot }$ yr^−1^ for the cooler K2 V star HD 189733, and Vidotto and Bourrier (2017) derived $\dot{M} \approx 1.2_{-0.75}^{+1.3}\times 10^{-15}~M_{\odot }$ yr^−1^ for the M2.5 dwarf GJ 436.

#### Slingshot Prominences

Jardine and Collier Cameron (2019) argued that prominences seen in absorption features when they transit in front of an active star can be signs of episodic mass loss like a wind. These prominences predominantly form at magnetic loop tops located around the co-rotation radius. Depending on the relative location of the Alfvén radius, these cooling loop-top condensations can break open and leave the corona as a wind. Extrapolation of the observed features to the entire star leads to estimates of $\dot{M}$ that seem to continue the trend for H wall estimates (see above), reaching $\dot{M}$ per unit surface area up to 3000 times the solar value. This study included three solar-type stars with masses of 0.82, 1.0, and 1.16 $M_{\odot }$ and two lower-mass M dwarfs.

#### Accretion Contamination in White Dwarf Atmospheres

This method is mentioned for completeness although it has focused on M dwarf winds. While white dwarf (WD) upper atmospheres should be made of pure H and He, contamination by heavier elements detected spectroscopically has been ascribed to accretion of interstellar gas. For close WD+M star binaries, Debes (2006) argued that the M dwarf wind accretes onto the WD, producing an equilibrium excess abundance of elements like Ca. Observations of several WD+M pairs would imply $\dot{M} \approx 10^{-16} - 6\times 10^{-15}~M_{\odot }$ yr^−1^ for the M dwarfs.

### Conclusions for Wind Mass-Loss Rates

Direct wind-detection methods use straightforward interpretation of observational data of emission of and absorption by the ionized stellar wind. Such effects tend to be weak so that essentially all observations have provided upper limits to $\dot{M}$. The most severe problem is competing and much brighter similar emission from the star itself. The upper limits from such observations are mostly in the range of $\dot{M} \approx 10^{-13} - 10^{-10}~M_{\odot }$ yr^−1^, and this includes solar-mass stars.

Indirect wind measurements use features that often “amplify” the evidence of a wind. The disadvantage of these methods is their need for complex models relating the observed features to the putative stellar winds. Such measurements have nevertheless been more successful in providing estimates for $\dot{M}$, mostly in the range of $\dot{M} \approx 10^{-16} - 10^{-12}~M_{\odot }$ yr^−1^.

**Fig. 2:**
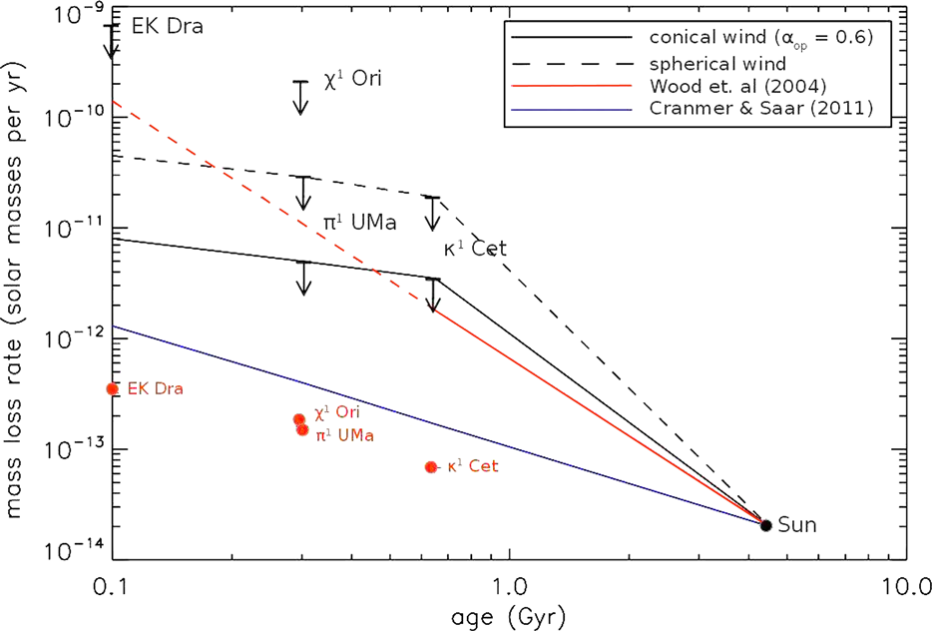
Mass-loss evolution for solar-type stars. The two black solid lines are upper limit estimates for the $\dot{M}$ evolution based on non-detections of stellar wind radio bremsstrahlung (Sect. 3.1; arrows indicate corresponding $\dot{M}$ upper limits). The upper black dashed line refers to a spherical wind, the lower solid black line to a conical wind with an opening angle of 40 degrees. The red circles are mass-loss estimates from the spin-down model of Johnstone et al. (2015) (Sect. 2), while the red line shows the fit from the Ly$\upalpha $ absorption model of Wood (2018) (Sect. 3.2; the dashed part is where Wood 2018 report a breakdown of the relation following observations). The blue solid line relates to the theoretical model of Cranmer and Saar (2011). (From Fichtinger et al. 2017.)

Collectively, the $\dot{M}$ estimates suggest a correlation between $\dot{M}$ and stellar activity, and therefore also rotation and age. The value of $\dot{M}$ most likely decays by 1–2 orders of magnitude during the evolution of a solar analog from a very active, early “Sun” with rapid rotation to a star like the present-day Sun (Fig. 2).

## Stellar High-Energy Radiation

All late-type main-sequence stars are X-ray emitters because of the presence of a hot, million-degree tenuous corona akin to the solar corona. The field of stellar coronal X-ray astronomy was opened after the first detailed study of the solar X-ray corona with Skylab, and the first stellar X-ray surveys with the *Einstein* satellite (Catura et al. 1975). It was soon realized that the Sun was among the weakest late-type stellar X-ray emitters in absolute terms, and that X-ray luminosities comprised at least the range of $10^{27} - 10^{30}$ erg s^−1^ and temperatures from the solar value, $\sim 2$ MK, to at least 10–20 MK.

As already mentioned in Sect. 1, activity is correlated with rotation and therefore also with age. This is due to the operation of a dynamo that amplifies magnetic fields in a process involving differential rotation and convection. These magnetic fields, in turn, can build up energy that they release in coronal heating, coronal mass ejections, and probably in wind heating and acceleration. This feedback loop leads to a convergence of the rotation period, and hence activity, about 700 Myr after the arrival on the ZAMS for a solar analog, regardless of the initial conditions. Before that age, activity evolution tracks are non-unique. The key relations are the following.

1) *Activity-rotation relation.* X-ray activity indicators such as the X-ray luminosity $L_{\mathrm{X}}$ (for a given stellar mass $M_{*}$), the X-ray surface flux, $F_{\mathrm{X}}$, or the ratio between total X-ray luminosity and bolometric luminosity $L_{\mathrm{bol}}$ scale with the stellar rotation period $P_{*}$ roughly as

11 $$ L_{\mathrm{X},\mathrm{ M_{*}}}, F_{X}, \frac{L_{X} }{L_{\mathrm{bol}}} \propto \Omega _{*}^{2} \propto P_{*}^{-2} $$

(e.g., Pallavicini et al. 1981; Pizzolato et al. 2003; Wright et al. 2011) but this holds only up to a threshold $\Omega _{*}$ above which these activity indicators saturate, i.e., become independent of $\Omega _{*}$. For solar analogs, this occurs at $\Omega _{\mathrm{sat}} \approx 15~\Omega _{\odot }$ (Johnstone et al. 2020).

2) *Rotation-age relation.* This was already discussed in Sect. 2. Skumanich (1972) proposed $\Omega \propto t^{-1/2}$ while Ayres (1997) found, for the equatorial rotation velocity $v$ of solar analogs,

12 $$ v \propto t^{-0.6} $$

and Johnstone et al. (2015) derived

13 $$ \Omega _{*} \propto t^{-0.57} $$

for solar-type stars.

3) *Activity-age relation.* This follows from the above two relations as, for example, ${L_{\mathrm{X}}/L_{\mathrm{bol}}} \propto t^{-1.2}$. Statistics from observations imply, for solar analogs,

14 $$ L_{\mathrm{X}} \approx (3\pm 1)\times 10^{28}t^{-1.5\pm 0.3}~\text{erg}\,\text{s}^{-1} $$

(Maggio et al. 1987; Güdel et al. 1997). Such relations would be important for research on the evolution of planetary atmospheres given the influence of X-rays on upper-atmospheric heating, ionization, chemistry, and loss into space. However, there are two important caveats to mention: i) Eq. (14) does not take saturation into account. ii) While approximately correct for ages exceeding ∼700 Myr for solar analogs, the extrapolation to younger stars is invalid given the wide range of initial $\Omega _{*}$ values observed after star formation. Eq. (14) must therefore not be used for solar-type stars with ages less than about 700 Myrs. Similar constraints apply to K and M dwarfs (Johnstone et al. 2015).

**Fig. 3:**
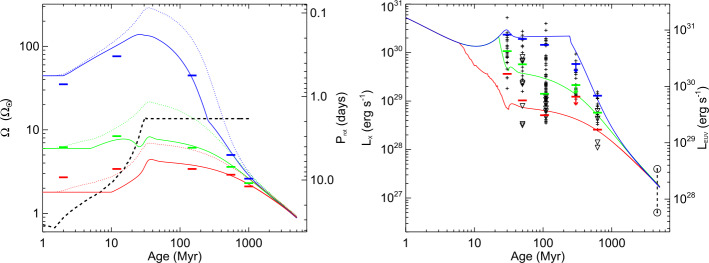
Left (**a**) Predicted rotational evolution tracks for stars at the 10th (red), 50th (green), and 90th (blue) percentiles of the rotational distribution. The solid and dotted lines show the envelope and core rotational evolution, respectively, and the horizontal solid bars show the observational constraints on the percentiles. The dashed black line shows the time dependent saturation threshold for $\dot{M}$, $B_{\text{dip}}$, and $L_{\text{X}}$. Right (**b**): Predicted $L_{\mathrm{X}}$ along each of the rotation tracks and comparisons to observed $L_{\mathrm{X}}$ values of single stars in several clusters with ages $\ge 30$ Myr (for observational samples at younger ages, see Johnstone et al. 2020), with upper limits shown by ▽ symbols. The solid horizontal bars show the 10th, 50th, and 90th percentiles of the observed distributions of $L_{\text{X}}$ at each age, calculated by counting upper limits as detections. The two solar symbols at 4.5 Gyr show the range of $L_{\text{X}}$ for the Sun over the course of the solar cycle. The scale on the right y-axis shows the associated $L_{\text{EUV}}$.

By combining rotational evolution models with Eq. (11), we find a range of evolutionary tracks for $L_{\mathrm{X}}$ (or the EUV luminosity $L_{\mathrm{EUV}}$), as illustrated in Fig. 3. The main points to emphasize are these: i) There is a wide spread of X-ray luminosities between the 10th and the 90th percentile of the initial rotation period distribution, starting at ages of 10–20 Myr and ending only at ages of ∼1 Gyr, when rotation converges. ii) The fastest rotators reside at an $L_{\mathrm{X}}$ saturation level for several 100 Myr, while the slowest rotators reach moderate X-ray luminosities already at a few tens of Myr. iii) Within the first few 100 Myr, $L_{\mathrm{X}}$ values are distributed across a factor of $\sim 40$ in $L_{\mathrm{X}}$, implying very different atmospheric evolution scenarios for planetary atmospheres due to the different $L_{\mathrm{X}}$ evolutionary tracks.

Tu et al. (2015) analyzed a large sample of $L_{\mathrm{X}}$ measurements from open clusters and compared it with the results implied from rotational evolution (Johnstone et al. 2015). They found, for the 10th (slowest), 50th, and 90th (fastest) percentile in the initial $\Omega _{*}$ distribution, corresponding to $\Omega _{0} = 1.8, 6.2$, and $45.6~\Omega _{\odot }$, respectively, the evolutionary trends

15 $$ L_{\mathrm{X}} = \left \{ \textstyle\begin{array}{l} 2.0\times 10^{31} t^{-1.12} \\ 2.6\times 10^{32} t^{-1.42} \\ 2.3\times 10^{36} t^{-2.50} \\ \end{array}\displaystyle \right . \quad L_{\mathrm{EUV}} = \left \{ \textstyle\begin{array}{ll} 7.4\times 10^{31} t^{-0.96} & \quad \text{10th percentile} \\ 4.8\times 10^{32} t^{-1.22} & \quad \text{50th percentile} \\ 1.2\times 10^{36} t^{-2.15} & \quad \text{90th percentile} \\ \end{array}\displaystyle \right . $$

($t$ in Myrs) where these approximations apply after the end of saturation at ages of $t_{\mathrm{sat}} = 5.7, 23$, and 226 Myrs, respectively. These evolutionary tracks are shown in Fig. 3 together with the corresponding rotation evolution tracks.

## Summary and Conclusions

The long-term evolution of magnetic fields, magnetic activity, high-energy emission and winds of stars on the main sequence are all intimately related to stellar spin-down through an internal magnetic dynamo. Since spin-down itself is due to removal of angular momentum in a magnetized wind, a feedback loop leads to convergence of rotation periods several hundred Myr after star formation. By implication, the winds and the high-energy radiation also decline with age and also converge to values almost only determined by age for a given stellar mass and ages beyond ∼1 Gyr. Some of these trends are observationally well studied while others are inferred from indirect observations. For the rotation rate $\Omega _{*}$, the wind mass-loss rate $\dot{M}$, and the X-ray luminosity $L_{\mathrm{X}}$, the following trends are found (mostly for solar-type stars):

$$\begin{aligned} \Omega (t) \propto & t^{-0.57} \quad {\mathrm{for}}~t>700~\text{Myr, from spin-down} \\ \dot{M}(t) \propto & t^{-1.75} \quad \text{for}~t>700~\text{Myr, from spin-down} \\ \dot{M}(t) \propto & t^{-2.33} \quad {\mathrm{for}}~t>700~\text{Myr, from Ly}\upalpha~\text{absorption} \\ L_{\mathrm{X}}(t) \propto & t^{-1.12} \quad \text{for}~t>5.7~\text{Myr, for slow rotator} \\ L_{\mathrm{X}}(t) \propto & t^{-1.42} \quad \text{for}~t>23~\text{Myr, for intermediate rotator} \\ L_{\mathrm{X}}(t) \propto & t^{-2.50} \quad \text{for}~t>226~\text{Myr, for fast rotator}. \end{aligned}$$

It should be understood that these are best-fit relations from various methods and observing data sets; the uncertainties in the above exponents may typically be of order several times 0.1. As indicated earlier, for a solar analog the age dependence for ages ≲700 Myr is non-unique as indicated above for $L_{\mathrm{X}}$; this is equally true for $\Omega $ and $\dot{M}$ as described in Sect. 2 and 3 but the relations above are given only for the ages ≳700 Myr.

These relations are important to study atmospheric evolution of planets, but even more important is a correct treatment of the evolution at ages ≲700 Myr for a solar analog when evolutionary tracks are non-unique and the initial conditions after the dispersal of the protoplanetary disk need to be taken into account as well.

Further high-energy properties of a star may matter. In particular, high-energy flares add considerably to the X-ray output of a star or may even be the principal contributors to such emission. Audard et al. (2000) found that the extreme-ultraviolet flare rate correlates linearly with the activity level represented by the average $L_{\mathrm{X}}$. Also, coronal mass ejections (CMEs) can complement if not dominate the mass-loss rate of a star although extrapolation of solar trends lead to excessive CME mass-loss rates, suggesting that CMEs must be suppressed at high activity levels (Drake et al. 2013).
